# Unprofessional Advertisements

**Published:** 1856-09

**Authors:** 


*Unprofessional Advertisements.*

We have received communications, from time to time, asking our opinion concerning the propriety of certain modes of advertising by physicians. Generally these questions have related to some speciality, such as "Diseases of the Eye," or "Diseases of the Chest," and we have preferred to let the parties interested form their own opinions, referring them always to the established Code of Ethics. But we have recently received a handbill, which purports to have been issued by Drs. Kreider & Kimber, of Prairie City. It is couched in language so perfectly resembling the ordinary quack advertisements of the newspapers, that we were very much surprised to see the name of Dr. H. W. Kreider connected with it.

The following constitutes a part of this handbill, viz.:—

"*Typhoid Fever.*—This much dreaded disease they are fully prepared to say that they can treat with universal success, having lost but three patients of this disease in nine years, out of several hundreds treated, and that many cases of this fever can be broken up in from three to six days, instead of allowing them to run from twenty to forty days.

"*Consumption.*—The extensive researches which have been instituted by the Medical Profession during the past few years, and which have resulted in bringing to light new and effectual remedies, warrant them in saying that Consumption, if taken in its earlier stages, *can be cured.* They are happy to inform those interested that they are fully prepared with instruments and other means necessary for determining as to whether the lungs are diseased or not, and if diseased, to what extent; and they are also prepared with necessary remedies for arresting such disease, unless it is far advanced.

"Fever Sores, Ulcers of long standing, Chronic Eruptions of the Skin, and various other Chronic Affections, such as Liver Complaint, Enlargement of the Spleen (commonly known as Ague Cake), Diseases of the Stomach, Scrofulous Affections, Diseases of Females, &c., &c.

"To the cure of these, their especial attention will be given, and which they are able to perform in the most successful and satisfactory manner."

When any regular graduate deliberately makes up his mind to commence such a system of advertising, he should precede the step by a return of his diploma to the institution from which it was obtained. The following is what the Code of Ethics, adopted by the American Medical Association, and by nearly all the State Medical Societies in the whole Union, says in relation to this subject. We commend it to the special attention of Drs. Kreider & Kimber:—

"SECTION 3. It is derogatory to the dignity of the Profession, to resort to public advertisements, or private cards, or handbills, inviting the attention of individuals affected with peculiar diseases—publicly offering advice and medicine to the poor gratis, or promising radical cures; or to publish cases and operations in the daily prints, or favor or encourage such publications, except in approved medical prints; to invite laymen to be present at operations; to boast of cures and remedies; to adduce certificates of skill and success, or to perform any other similar acts. These are the ordinary practices of empirics, and are highly reprehensible in a regular physician."

